# Comparative analysis of deep learning architectures in solar power prediction

**DOI:** 10.1038/s41598-025-14908-x

**Published:** 2025-08-28

**Authors:** Montaser Abdelsattar, Mohamed A. Azim, Ahmed AbdelMoety, Ahmed Emad-Eldeen

**Affiliations:** 1 Electrical Engineering Department, Faculty of Engineering, South Valley University, Qena, 83523 Egypt; 2 Department of Computer Science, College of Computer and Cyber Sciences, University of Prince Mugrin, Medina, 42241 Saudi Arabia; 3 Renewable Energy Science and Engineering Department, Faculty of Postgraduate Studies for Advanced Sciences (PSAS), Beni-Suef University, Beni-Suef, 62511 Egypt

**Keywords:** Solar forecasting, Deep learning, Renewable energy, Energy prediction, Engineering, Electrical and electronic engineering

## Abstract

Integrating renewable energy sources into the electricity grid requires accurate forecasts of solar power production. With the aim of enhancing the accuracy and reliability of forecasts, this study presents a comprehensive comparative analysis of eight state-of-the-art Deep Learning (DL) architectures—Autoencoder, Long Short-Term Memory (LSTM), Gated Recurrent Unit (GRU), Simple Recurrent Neural Network (SimpleRNN), Convolutional Neural Network (CNN), Temporal Convolutional Network (TCN), Transformer, and Lightweight Informer for Long Sequence Time-Series Forecasting (InformerLite)—applied to solar power prediction using a dataset with 4,200 historical records and 20 meteorological and astronomical features. A comprehensive assessment of Root Mean Squared Error $\:\left(\varvec{R}\varvec{M}\varvec{S}\varvec{E}\right)$, Mean Absolute Error $\:\left(\varvec{M}\varvec{A}\varvec{E}\right)$, Mean Absolute Percentage Error $\:\left(\varvec{M}\varvec{A}\varvec{P}\varvec{E}\right)$, and Coefficient of Determination $\:\left({\varvec{R}}^{2}\right)$ metrics was performed on the training, validation, and test datasets. The TCN model had the greatest performance across all models, achieving a test R² of 0.7786, an $\:\varvec{R}\varvec{M}\varvec{S}\varvec{E}$ of 429.4863, and a balanced relative standard deviation ($\:\varvec{R}\varvec{S}\varvec{D}$) of 0.6827, so exhibiting an exceptional capacity to capture temporal patterns. The Autoencoder achieved a $\:{\varvec{R}}^{2}$ of 0.7648 and had the greatest overall performance on the entire dataset, resulting in a Whole $\:{\varvec{R}}^{2}$ of 0.8437. In contrast, the Transformer model demonstrated significantly poorer performance (Test $\:{\varvec{R}}^{2}$ = 0.0714), underscoring its limitations in this context without any architectural modifications. This study not only demonstrates the best DL models for solar power forecasting as qualified by useful statistical metrics, but also provides a scalable, interpretable, and extensible forecasting framework for real-world energy systems. The findings verify the informed DL integration to smart grid scenarios, laying the foundations for further developments in hybrid modeling, multi-horizon prediction, and deployment in resource-constrained environments with limited computational power and resources.

## Introduction

The continuously increasing world-wide demand for alternative sources of clean and sustainable energy has placed a bigger emphasis on the accurate forecasting of solar electricity production. One extremely exciting renewable energy source that requires precise prediction models to be included in the energy system as best as possible, enhance energy management, and ensure dependability is solar energy. Traditional time series forecasting methods fail to model well the complex, non-linear patterns present in solar power data^1–3^. As such, using advanced Deep Learning (DL) techniques to improve prediction accuracy has attracted growing attention. The purpose of this study is to evaluate in forecasting solar power generation the efficiency of eight DL algorithms: Lightweight Informer for Long Sequence Time-Series Forecasting (InformerLite), Long Short-Term Memory (LSTM), Autoencoders, Gated Recurrent Unit (GRU), Recurrent Neural Network (RNN), Transformer, Convolutional Neural Network (CNN), and Temporal Convolutional Network (TCN). Several of these models, particularly sequence-based architectures such as LSTM, GRU, RNN, and TCN, have demonstrated strong capabilities in capturing temporal dependencies and non-linear interactions within large-scale time series datasets^4^. The study aims to identify the most reliable and accurate model for solar power forecasting by comparing several methods, thereby advancing renewable energy technology and its application in sustainable energy systems.

Precise prediction of solar power generation is essential for several reasons. First and foremost, it facilitates the seamless incorporation of solar energy into the electrical grid, thus aiding in the equilibrium of energy supply and demand and diminishing dependence on non-renewable energy sources. Accurate predictions, enable grid managers to properly control the energy storage systems, which reduce impacts due to the solar energy variations^5^. Furthermore, a reliable and accurate prediction is of great importance to ensure the financial profitability of solar energy by increasing the performance and controllability of solar parks. Thus, although operating expenses of the company have dropped, investment income has grown^6^. Reliable projections also facilitate the establishment of well-considered and knowledgeable judgments on next solar energy projects, so promoting the switch to renewable and sustainable energy sources. Effective assessments of solar energy generation, as a result, can help decrease the risk of power interruption and establish an available power supply by enhancing power system stability and reliability to the grid. Thus, to help meet global energy targets, support the environmental accountability of developers, and foster the technological advancements for renewable energy, enhancing the solar energy yield prediction capability is of paramount importance.

In solar power forecasting, DL has evolved into a powerful tool with clear benefits over more traditional methods. Sometimes the complex, nonlinear links and temporally dependent patterns in solar power data are too difficult for conventional statistical models to adequately depict. Conversely, DL algorithms excel in these fields and consequently have rather great success in time-series prediction. Using large datasets not readily visible with conventional approaches allows DL models to discover intricate patterns and trends^7–9^.

An accurate estimate of solar power output is crucial for achieving the highest level of integration of solar energy into the power system. However, due to the intricate and nonlinear characteristics of solar data, which are impacted by various climatic and environmental factors, existing forecasting algorithms are unable to accurately predict solar power output. Inappropriate grid management, higher running costs, and less reliability of solar power plants follow from this disparity. Finding and appreciating the best DL techniques for handling complex solar power data and generating accurate forecasts is crucial^10^.

The application of Machine Learning (ML) and DL in Photovoltaic (PV) systems has improved the performance, reliability, and predictability of solar energy applications. ML methods are everywhere utilized to accurately predict solar power generation and ambient conditions influencing PV yield^11–13^. On the other hand, DL approaches have proved to be highly successful for automation problems, for example defect detection in solar cells^14^, fault detection and performance predication^15^, surface condition monitoring via image classification^16^. In addition, advanced image process and computer vision techniques have allowed for accurate PV panel damage detection which further facilitates predictive maintenance and operating optimization^17^. These developments highlight the importance of smart algorithms in making PV systems smarter, more adaptive and more robust energy conversion tools.

The development of DL has boosted the credibility and accuracy of solar power prediction, mainly in fields like Electric Vehicle (EV) battery swap station, and integrated grid connection of renewable energy. For example, the application of LSTM models to forecast the solar power availability in EV swapping stations can contribute to an optimal scheduling of the battery charging, ultimately decreasing the reliance on the grid and improving energy efficiency^18^. DL techniques, including LSTM, AutoRegressive Integrated Moving Average (ARIMA), and Dual Attention-based RNNs, have been evaluated for solar irradiance forecasting, with LSTM models exhibiting enhanced performance in error reduction and real-time application^19^. Although solar forecasting is a primary emphasis, the application of ML in infrastructure monitoring has also gained traction. Research employing magnetostrictive sensors alongside decision trees and neural networks has attained elevated classification accuracy for bridge health evaluation, demonstrating the adaptability of ML in sensor-driven predictive modeling^20^. The amalgamation of Random Forest (RF) and Deep Neural Network (DNN) with frequency domain data has facilitated real-time structural integrity monitoring in prototype beam bridges, demonstrating the wider application of these methods beyond energy sectors^21^.

Recent hybrid architectures, which combine recurrent with attention-based mechanisms, such as the Transformer-Infused Recurrent Neural Network (TIR), have proved effective in maintaining data complexity and temporal dependence^22^. Ensemble learning methods have therefore recently been attracting significant attention, such as Stack-based Ensemble Fusion with Meta-Neural Network (SEFMNN) and Extreme Gradient Boosting-Stacked Ensemble (XGB-SE), which have obtained state-of-the-art results in different regions by combining a variety of base-learners^23^. In addition, decomposition methods for example Ensemble Empirical Mode Decomposition (EEMD) have also been widely used for enhancing model interpretability and prediction accuracy by extracting the intrinsic signal modes before inputting them to neural architectures including LSTM and Artificial Neural Network (ANN)^24^. Recent reviews have clarified the transformative effects of DL and ML technologies have on solar forecasting and potential to counter nonlinearities and uncertainties found in solar irradiance data, which in turn can help improve grid reliability and sustainable energy planning^25^. Additionally, recent advancements in modified ANN structures and lightweight Gradient Boosting Machine (GBM) structures such as Regularized Lightweight Artificial Neural Network (RELAD-ANN) and Light Gradient Boosting Machine (LightGBM) also provide plausible ways to combine computational complexity and predictive performance for real-time world solar energy systems^26^.

This study aims to systematically evaluate the prediction of solar power output using multiple advanced DL algorithms. The particular aim of the study is to assess the accuracy of eight DL models—Autoencoders, GRU, RNN, LSTM, Transformer, CNN, TCN, and InformerLite—in forecasting solar power generation. By means of important performance criteria like Coefficient of Determination (R²) scores, Root Mean Squared Error (RMSE), Mean Absolute Error (MAE), Mean Absolute Percentage Error (MAPE), mean, Standard Deviation (SD), and Relative Standard Deviation (RSD) of the predictions generated on the test set, the study aims to evaluate the precision and dependability of these models. The goal of this work is to identify the most suitable DL algorithm for solar power generation prediction. This involves analyzing the models’ ability to faithfully depict complex time-based patterns and nonlinear linkages within the data. Moreover, the study also seeks to deliver actionable insights into the strengths and limitations of each model in the context of renewable energy forecasting, thereby supporting the integration of solar power into the energy grid. Through better understanding of how DL may improve solar power forecasting, the study advances more reliable and effective renewable energy systems. Finally, reaching these targets will offer important new perspectives on the field of renewable energy forecasting, thereby supporting better decision-making and solar power generation optimization.

**Fig. 1:**
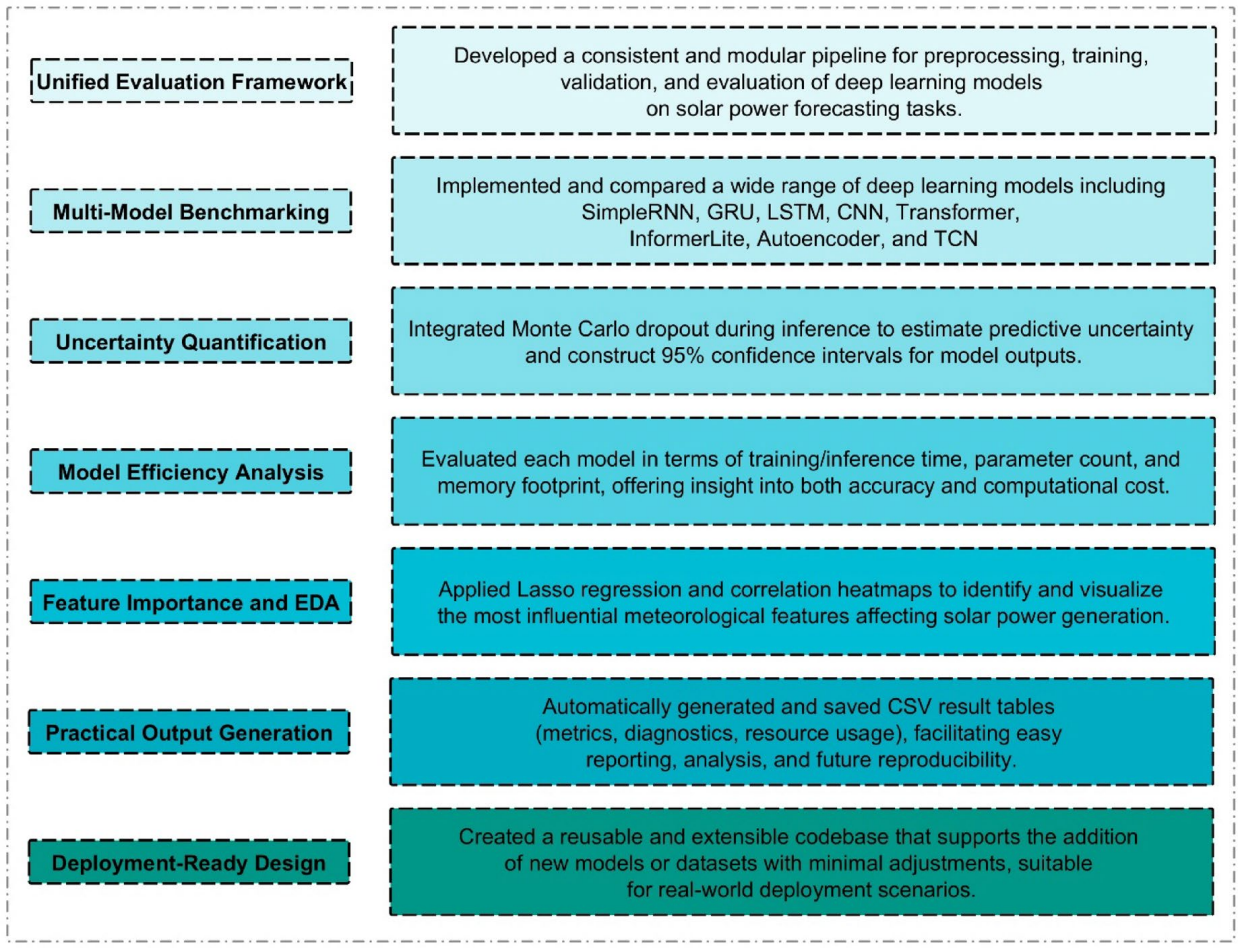
Key Contributions of the Proposed Solar Power Forecasting Framework.

The objectives of the proposed research include the development of a robust and scalable model for accurate solar power prediction using state-of-the-art DL techniques. As shown in Fig. 1, a major contribution of this work is to extend beyond the conventional “Model Training” approach, testing a broad range of neural network architectures, judging them not only on predictive performance but also based on efficiency and deployability. It is intended to be extensible and modular in nature for example researchers should be able to easily add new models and types of data. Moreover, practical aspects such as uncertainty estimation and automatic result generation are emphasized, making the solution viable for real-world energy systems.

## Methodology

### Data presentation

In this study, this dataset comprises 4200 samples of historical records of solar power generation, and for each record, each annotated with total of 20 meteorological and astronomical input features and as well as one target output, the generated power in kilowatts. The input parameters include the temperature, humidity, pressure, precipitation, cloud cover at multi-levels altitude, solar radiation, wind level at different heights, and pressure levels, solar angle of incidence, solar position angle such as zenith and azimuth.

**Fig. 2:**
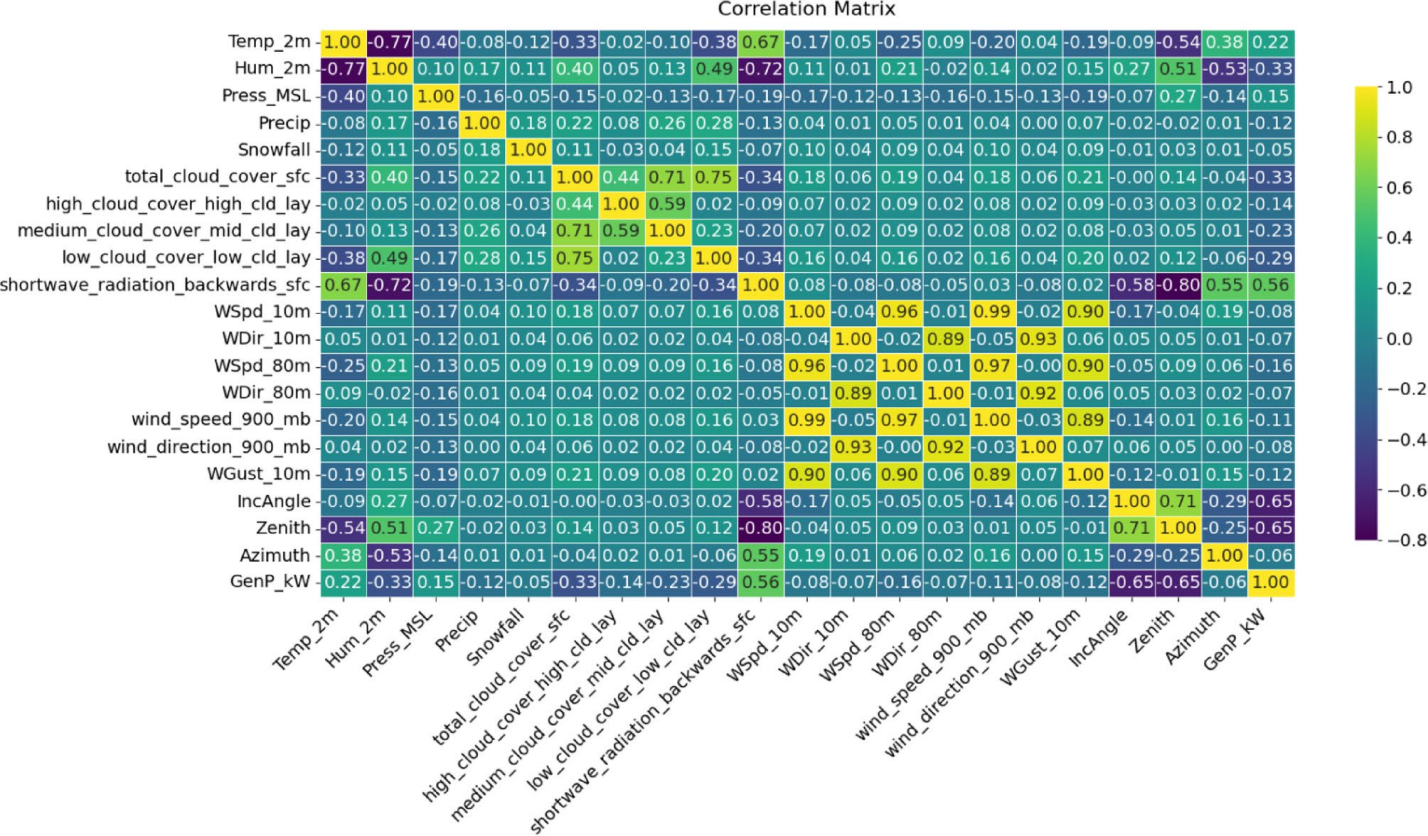
Correlation matrix showing relationships between various features in the dataset.

To analyze inter-feature relationships, this study calculated a correlation matrix as shown in Figure 2. This heatmap illustrates the positive or negative linear correlations between the variables. Shortwave radiation and zenith is correlated most strongly with the output power with the other variables of humidity and azimuth also having moderate correlation. Strong inter-correlations across different wind layers were evident, indicating redundancy that could be reduced by selecting of features.

**Fig. 3:**
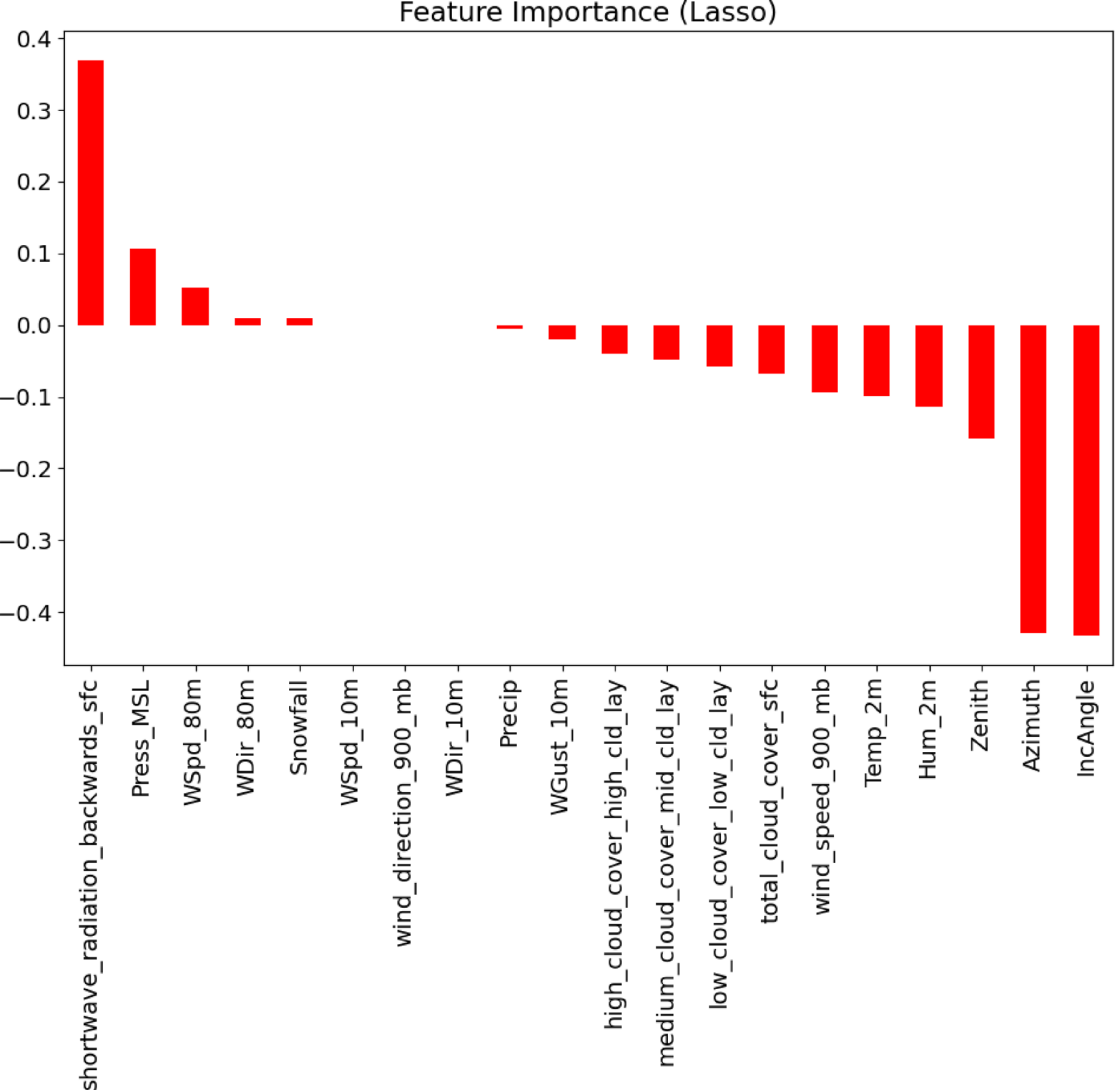
Lasso Regression-Based Feature Importance for Solar Power Forecasting.

Besides correlation analysis, Lasso regression was used to perform feature selection and to measure the importance of the independent variables against the target output. A resulting plot of feature importance is displayed in Fig. 3 and shortwave radiation is identified as the most indicative feature value, and then mean sea level pressure, wind speed at 80 m, and wind direction at 80 m also are influential. In contrast, the angle of incidence and azimuth were penalized to very low coefficients in practise, meaning that the angle of incidence and azimuth only weakly contributed to the predictive model under the sparsity constraint of the Lasso. These findings informed the future design of model inputs, allowing the elimination or reduction of low-impact characteristics to optimize training and enhance generalization.

### Deep learning algorithms

**Fig. 4:**
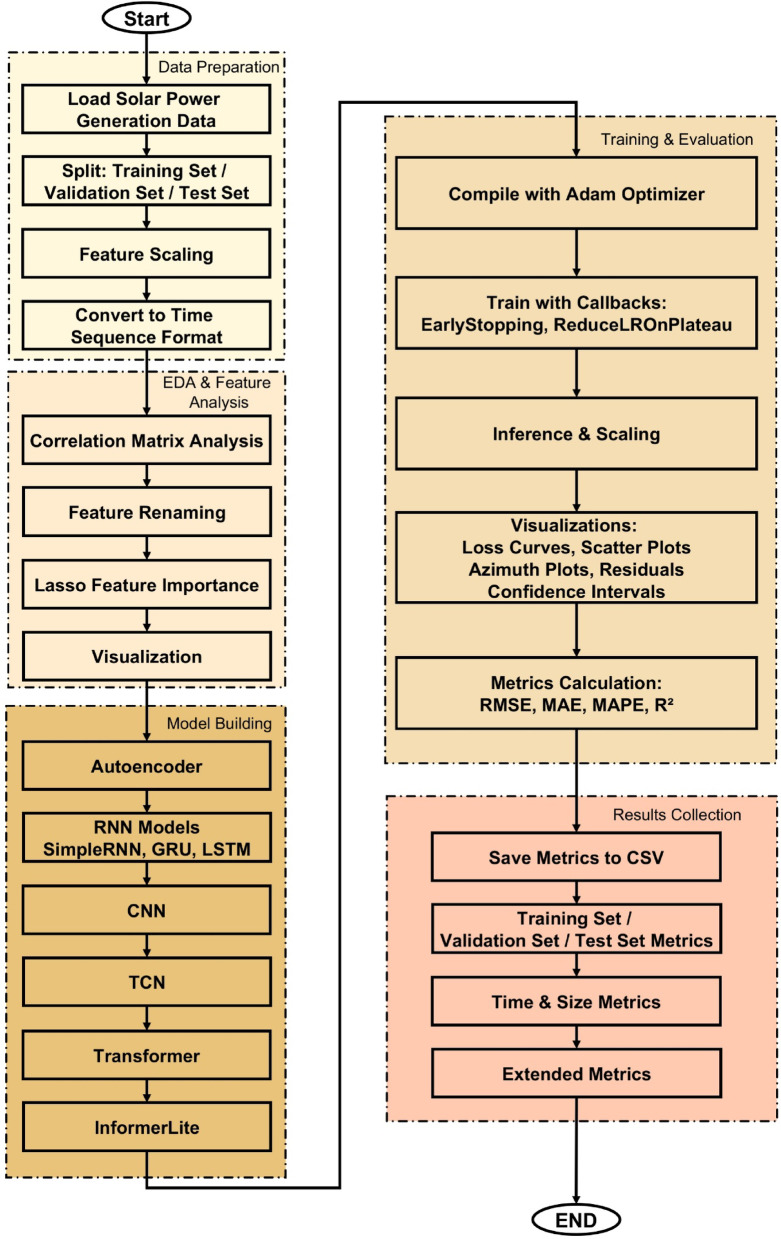
Flowchart of the Proposed Solar Generation Prediction Framework Using Deep Learning Algorithms.

An evaluation was performed to compare the predictive power of a few DL models in the estimation of solar PV power production. The proposed approach incorporates robust data pre-processing, an exploratory analysis, and several DL techniques to provide accurate solar power generation predictions. The end-to-end system is shown in Fig. 4.

#### Data Preparation

The work-flow starts by uploading a pre-processed dataset of historical solar plants generation as well as correlated meteorological variables. The dataset is split into training, validation and test sets in the ratio of (70:20:10) % to maintain neutrality while calculating the scores. The input space will be normalized with standardization through feature scaling. The data is restructured into time-sequence for modeling sequences with their temporal dependencies.

#### Exploratory data analysis and feature engineering

Exploratory Data Analysis (EDA) is performed prior to model estimation by a correlation matrix analysis which provides insight into the relationships between features and suggests feature selection. The variable names are recoded to have long names for the sake of intelligibility. Lasso regularization is used for feature importance quantification and dimensionality reduction. The interpretations are visualized to help understanding and interpretation of the model decisions.

#### Model Building

A broad array of DL models is employed to assess the efficacy of different architectures in predicting solar power generation. The configuration features an Autoencoder, designed as a dense feedforward network with a bottleneck layer to acquire compact latent representations of the input characteristics. RNNs, namely Simple Recurrent Neural Network (SimpleRNN), GRU, and LSTM, are used to capture temporal correlations in data because to their memory-based architecture. CNNs use one-dimensional convolutional layers to extract localized temporal patterns. The TCN enhances temporal learning by using dilated causal convolutions and skip connections, which aids in the detection of long-range temporal patterns. The Transformer can handle complex relationships and take sequences into consideration at the same time due to its self-attention mechanisms. InformerLite, a lightweight and efficient variant of Transformer, is well matched to the time series forecasting task. All models are implemented with the Keras Application Programming Interface (API), backend TensorFlow 2.16, and as compared to this model, enhanced compatibility, scalability, and hardware acceleration support are guaranteed.

#### Training and evaluation

Models are constructed with the Adam optimizer and trained with early stopping and learning rate reduction callbacks. After learning, the inference is performed on all the datasets. Predictions are transformed back to the original scale. Evaluation metrics include RMSE, MAE, MAPE, and R^2^ are computed on training, validation, and test sets. Several visualizations — including loss curves, scatter plots, solar azimuth comparisons, residual histograms, and Confidence Intervals (CIs) — are produced for visual validation.

During testing Monte Carlo dropout is used to calculate uncertainty intervals (95% confidence), contributing to the interpretability of the model.

#### Result collection

All metrics are stored in a uniform format as CSV files for reproducibility and downstream analysis. Other third-party tests for residual behavior, such as Shapiro–Wilk, Jarque–Bera, and Ljung–Box (if implemented) are also applied. This work also reports model size, training/inference time, and RSD of test predictions for a more complete comparison.

Figure 4 provides a complete overview of the entire methodology pipeline, from raw data ingestion to model evaluation and result export.

#### Experimental setup and configuration parameters

The experimental design of this work was meticulously organized to guarantee uniformity, repeatability, and dependable model assessment. The solar power generation recovery dataset was preprocessed with standard normalization and divided into training, validation and test sets in a stratified manner. This work trained multiple models with various DL architectures and fixed learning rate Adam optimization method with early stopping to avoid overfitting. To evaluate the performance, this research used RMSE, MAE, MAPE, and R² measures over various data partitions. Furthermore, this research also used Monte Carlo dropout for predicting uncertainty. Table 1 gives a comprehensive overview of the major parameters applied in this study, including the configuration of the data processing, the choice of the model, the options in the training, and the diagnostic tests.

**Table 1:** Summary of experimental parameters and model configuration.

Category	Parameter	Value / Setting
**General Setup**	Random Seed	42
	Framework	Keras 3 with TensorFlow 2.16
	Dataset File	“Dataset.csv”
**Data Processing**	Train / Validation / Test Split	70% / 20% / 10% (via 30% split then 1/3 split of temp set)
	Feature Scaling Method	StandardScaler (z-score normalization)
	Time Series Conversion	Reshaping input as sequences with shape (features, 1)
**Feature Engineering**	Feature Renaming	Applied for clarity (e.g., temperature_2_m_above_gnd → Temp_2m)
	Feature Selection	Lasso Regression ( alpha = 0.001 )
**Optimization**	Optimizer	Adam
	Learning Rate	0.001
	Loss Function	Mean Squared Error (MSE)
	Early Stopping	patience = 10 epochs, restore best weights
	Learning Rate Scheduler	ReduceLROnPlateau, patience = 5 epochs
**Training Configuration**	Epochs	200 (with early stopping)
	Batch Size	32
**Evaluation Metrics**	Forecast Metrics	RMSE, MAE, MAPE, R²
	Diagnostic Tests	Shapiro-Wilk, Jarque-Bera, Ljung-Box
	Uncertainty Estimation	Monte Carlo Dropout (100 runs)
**Visualization**	Plots	Loss curves, predicted vs. real scatter, azimuth plots, residual histograms, 95% CIs
**Models Evaluated**	Autoencoder	Dense + Bottleneck + Reconstruction
	RNN Models	SimpleRNN, GRU, LSTM
	CNN	1D Conv + MaxPooling + Global Avg Pooling
	TCN	Dilated Causal Convolutions
	Transformer	Multi-Head Attention blocks with feedforward layers
	InformerLite	Causal Conv + Attention + Global Avg Pooling

### Evaluation metrics

A composite accuracy measure consisting of accuracy, reliability, and statistical consistency measures was adopted to provide a complete characterization to the forecasting performance of the proposed DL models for solar power generation. Equation (1), Eq. (2), and Equation (3) were applied to the aggregate data, training data, and test data to compute R^2^ for the aggregate, training and test data sets respectively. These scores quantify the proportion of variance in the observed data that is explained by the predictions. To assess prediction accuracy more concretely, standard error-based metrics were applied. The RMSE, which penalizes larger deviations, is presented in Eq. (4). The MAE, which measures the average magnitude of errors without considering their direction, is formulated in Eq. (5). Similarly, Eq. (6) defines the MAPE, a relative metric that expresses errors as a percentage of the actual values. To enhance comprehension of the distributional characteristics of model outputs on the test set, the Mean of Test Predictions is determined as illustrated in Eq. (7), and its SD is derived using Eq. (8). Finally, Eq. (9) delineates the RSD, which provides a standardized measure of variability in relation to the mean. Collectively, these criteria offer a thorough assessment of predictive performance concerning accuracy, stability, and generality.

1 $$\:{R}_{whole}^{2}=1-\frac{{\sum\:}_{i=1}^{n}{({y}_{i}-{\widehat{y}}_{i})}^{2}}{{\sum\:}_{i=1}^{n}{({y}_{i}-\stackrel{̄}{y})}^{2}}$$

2 $$\:{R}_{\text{t}\text{r}\text{a}\text{i}\text{n}}^{2}=1-\frac{{\sum\:}_{i=1}^{{n}_{train}}{({y}_{train,\:i}-{\widehat{y}}_{train,\:i})}^{2}}{{\sum\:}_{i=1}^{{n}_{train}}{({y}_{train,\:i}-{\stackrel{̄}{y}}_{train})}^{2}}$$

3 $$\:{R}_{\text{t}\text{e}\text{s}\text{t}}^{2}=1-\frac{{\sum\:}_{i=1}^{{n}_{test}}{({y}_{test,\:i}-{\widehat{y}}_{test,\:i})}^{2}}{{\sum\:}_{i=1}^{{n}_{test}}{({y}_{test,\:i}-{\stackrel{̄}{y}}_{test})}^{2}}$$

4 $$\:RMSE=\sqrt{\frac{1}{n}{\sum\:}_{i=1}^{n}{({y}_{i}-{\widehat{y}}_{i})}^{2}}$$

5 $$\:MAE=\frac{1}{n}{\sum\:}_{i=1}^{n}\left|{y}_{i}-{\widehat{y}}_{i}\right|$$

6 $$\:MAPE=\frac{100}{n}{\sum\:}_{i=1}^{n}\left|\frac{{y}_{i}-{\widehat{y}}_{i}}{{y}_{i}}\right|$$

7 $$\:Mean\:of\:Test\:Set=\frac{1}{{n}_{test}}\sum\:_{i=1}^{{n}_{test}}{\widehat{y}}_{test,i}$$

8 $$\:{SD}_{test}=\sqrt{\frac{1}{{n}_{test}}\sum\:_{i=1}^{{n}_{test}}{({\widehat{y}}_{test,\:i}-{\stackrel{̄}{y}}_{test})}^{2\:}}$$

9 $$\:RSD=\frac{Standard\:Deviation\:of\:Test\:Set}{Mean\:of\:Test\:Set}$$

## Results

To evaluate the performance of a number of advanced DNN models for predicting solar power generation, this study performed extensive experiments with a variety of architectures. Model performance was tested on three sets: training, validation, and testing. RMSE, MAE, MAPE, R² were accepted as standard regression measures of performance. In addition, uncertainty quantification and residual analysis were carried out for diagnostic purposes.

### Model complexity and efficiency

Table 2 delineates the intricacies of model complexity and runtime. The Autoencoder had the highest computational efficiency, characterized by the minimal parameter count and compact model size (3,537 parameters, approximately 75 KB), with the briefest training duration (around 5.7 s). Conversely, the Transformer and TCN, despite their greater complexity (93,574 and 86,913 parameters respectively), need considerably longer training durations (~ 17.8s and ~ 32.1s).

**Table 2:** Model complexity and runtime metrics sorted by training duration (Seconds).

Model	Parameters	Model Size (KB)	Training Time (s)	Inference Time (s)
Autoencoder	3537	75.8096	5.6740	0.0183
SimpleRNN	4289	75.9639	8.3670	0.1554
Transformer	93,574	1212.7295	17.8085	0.7601
LSTM	16,961	224.5088	20.3755	0.2510
InformerLite	29,313	396.6299	21.4353	0.1641
CNN	6465	108.0205	21.6511	0.0401
GRU	12,929	177.2500	25.5519	0.2741
TCN	86,913	1132.4961	32.0912	0.4429

### Model training and convergence behavior

**Fig. 5:**
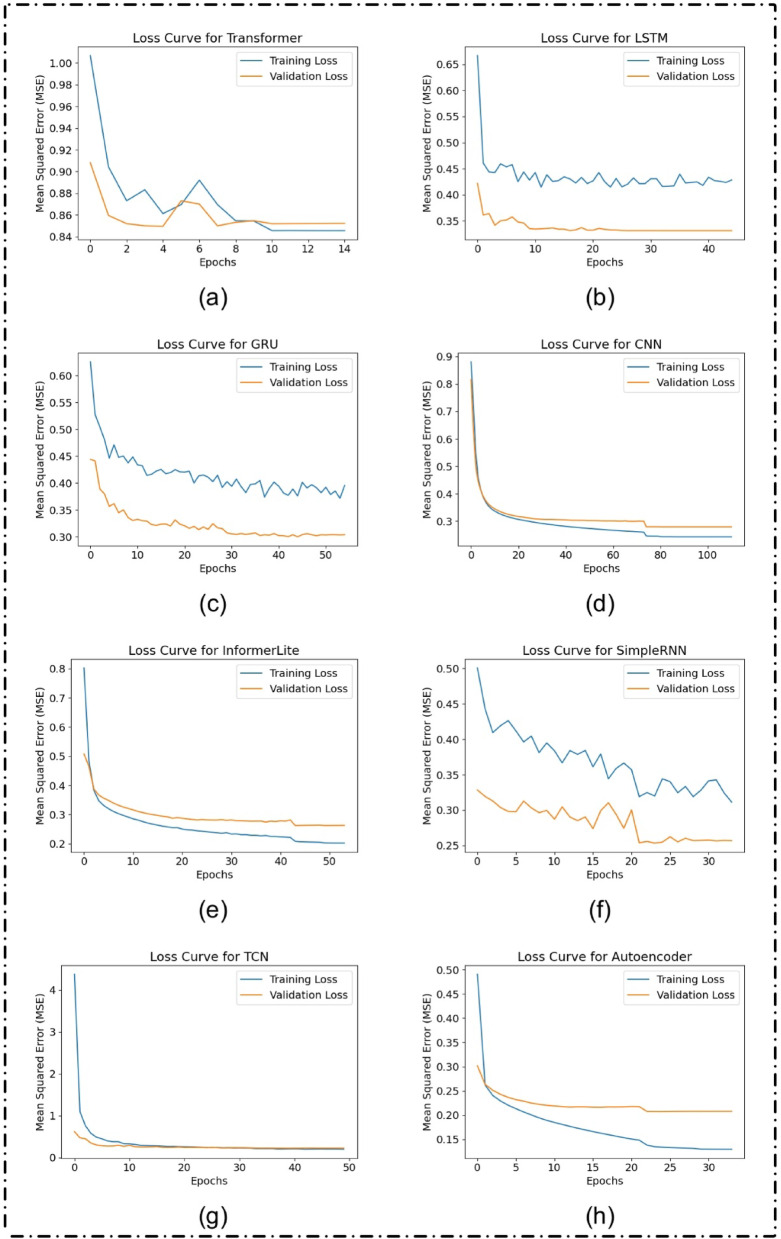
Training and validation loss curves (Mean Squared Error) for various deep learning architectures applied to solar power forecasting: (**a**) Transformer, (**b**) LSTM, (**c**) GRU, (**d**) CNN, (**e**) InformerLite, (**f**) SimpleRNN, (**g**) Temporal Convolutional Network (TCN), and (h) Autoencoder.

Figure 5 shows the MSE loss of each model on training and validation. From the models, among the models Autoencoder and TCN showed the stable and faster convergence. On the other hand, the Transformer model had large validate loss implying overfitting or not enough feature extraction in this domain.

### Prediction accuracy on training data

**Fig. 6:**
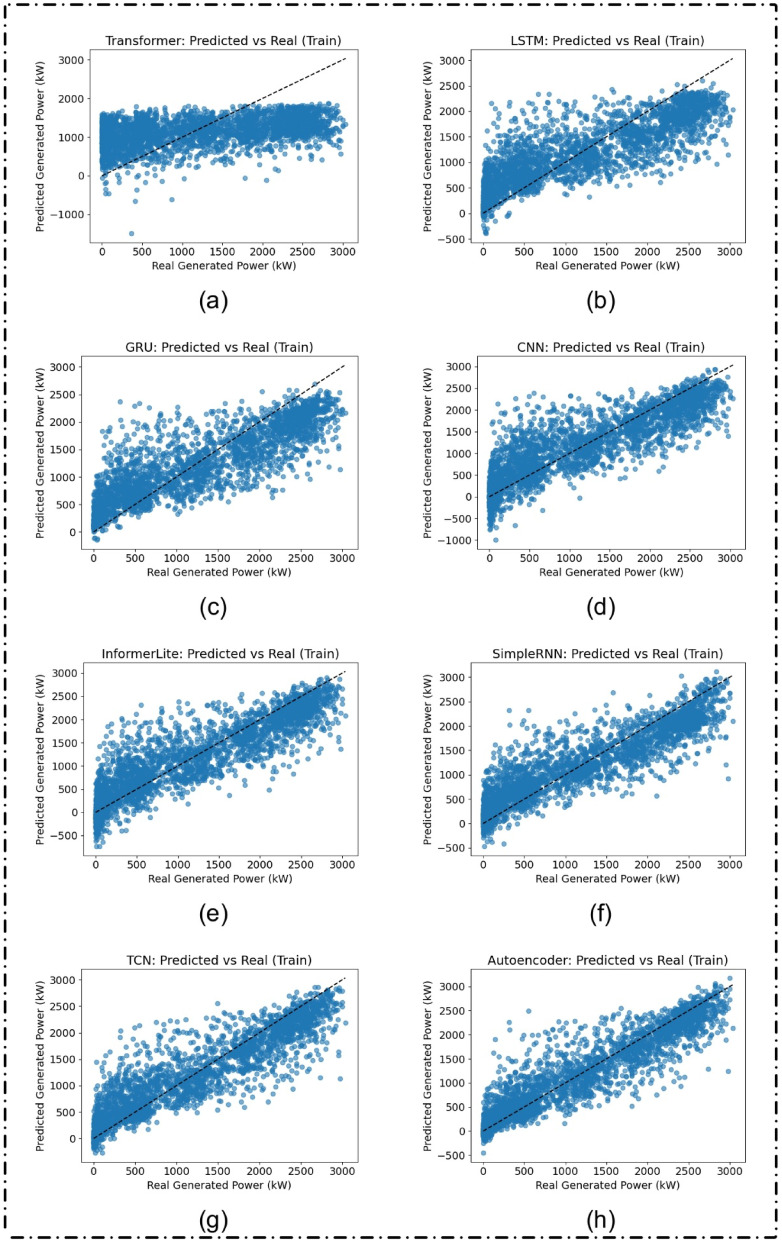
Predicted versus actual solar power generation (in kW) on the training dataset for different deep learning models: (**a**) Transformer, (**b**) LSTM, (**c**) GRU, (**d**) CNN, (**e**) InformerLite, (**f**) SimpleRNN, (**g**) Temporal Convolutional Network (TCN), and (**h**) Autoencoder.

Figure 6 illustrates the prediction performance of the models on the training set. The Autoencoder, TCN, and SimpleRNN models exhibited a high correlation between anticipated and actual values, signifying robust learning capability. Table 3 corroborates this, indicating that the Autoencoder attained the greatest R² of 0.8677, with the TCN closely following at R² = 0.8374. Conversely, the Transformer had a markedly worse performance, achieving a R² of just 0.1551.

**Table 3:** Evaluation metrics on training set ordered by increasing R² (Lower to higher Efficiency).

Model	RMSE	MAE	MAPE	*R*²
Transformer	862.0321	745.9043	148581.6866	0.1551
LSTM	525.9054	418.8194	25620.8813	0.6855
GRU	486.6311	385.2489	24411.0665	0.7307
CNN	462.4756	345.9687	18104.5631	0.7568
InformerLite	423.7508	313.7759	30930.4242	0.7958
SimpleRNN	410.9217	315.5270	21515.0806	0.8080
TCN	378.1977	270.5365	11125.2028	0.8374
Autoencoder	341.0466	234.7443	13257.2408	0.8677

### Generalization to validation set

**Fig. 7:**
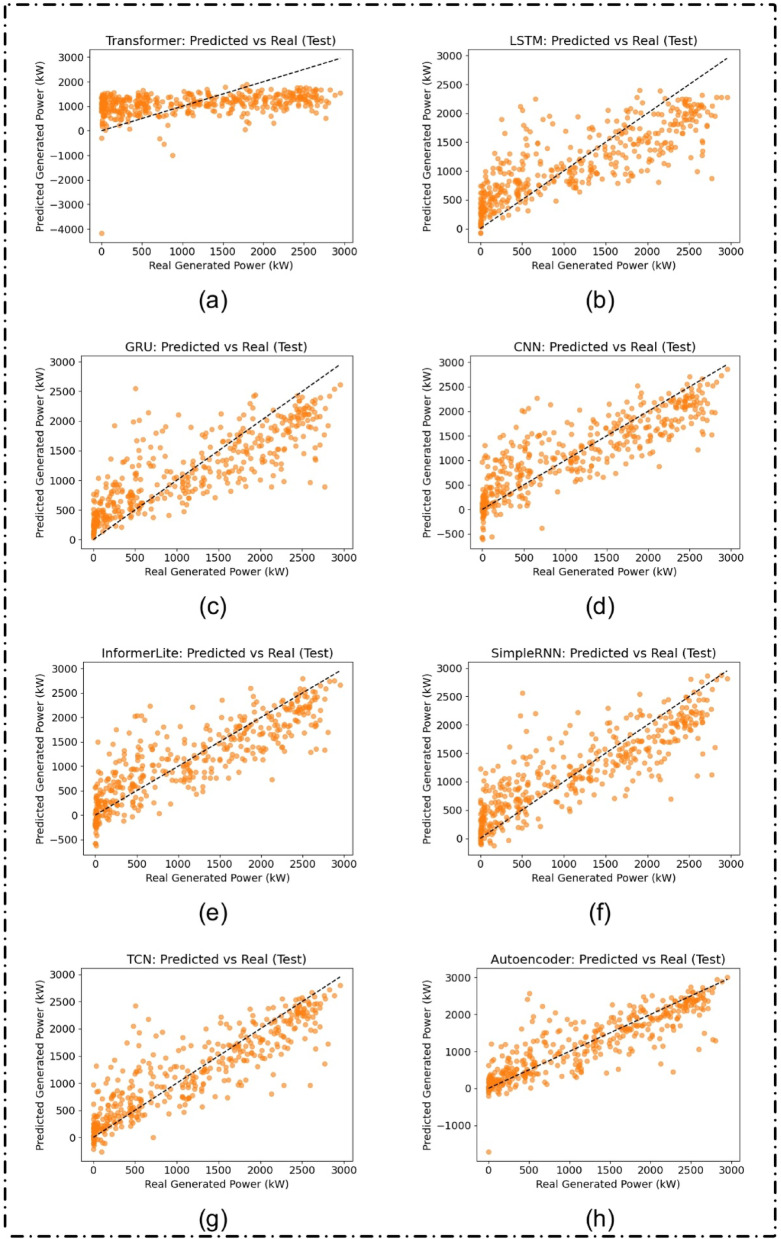
Predicted versus actual solar power generation (in kW) on the test dataset for different deep learning models: (**a**) Transformer, (**b**) LSTM, (**c**) GRU, (**d**) CNN, (**e**) InformerLite, (**f**) SimpleRNN, (**g**) Temporal Convolutional Network (TCN), and (**h**) Autoencoder.

For comparisons the model predictions on the test set are depicted in Fig. 7, revealing that models such as the Autoencoder and TCN did well on data it had experienced before. This is reinforced by Table 4, it is shown that the Autoencoder obtains R² of 0.7978 and the TCN follows with 0.7853. These results demonstrate strong generalization.

**Table 4:** Evaluation metrics on validation set ordered by increasing R² (Lower to higher Efficiency).

Model	RMSE	MAE	MAPE	*R*²
Transformer	864.4696	755.1639	101714.3527	0.1712
LSTM	539.8949	427.3706	33203.2621	0.6767
GRU	513.7007	394.8666	33446.1366	0.7073
CNN	495.7869	370.4419	16653.6720	0.7274
InformerLite	480.1903	351.7369	39847.8133	0.7443
SimpleRNN	472.0302	355.3144	21852.0857	0.7529
TCN	439.9661	300.8601	15749.0408	0.7853
Autoencoder	427.0279	285.8551	6525.7144	0.7978

### Azimuthal feature interpretability

**Fig. 8:**
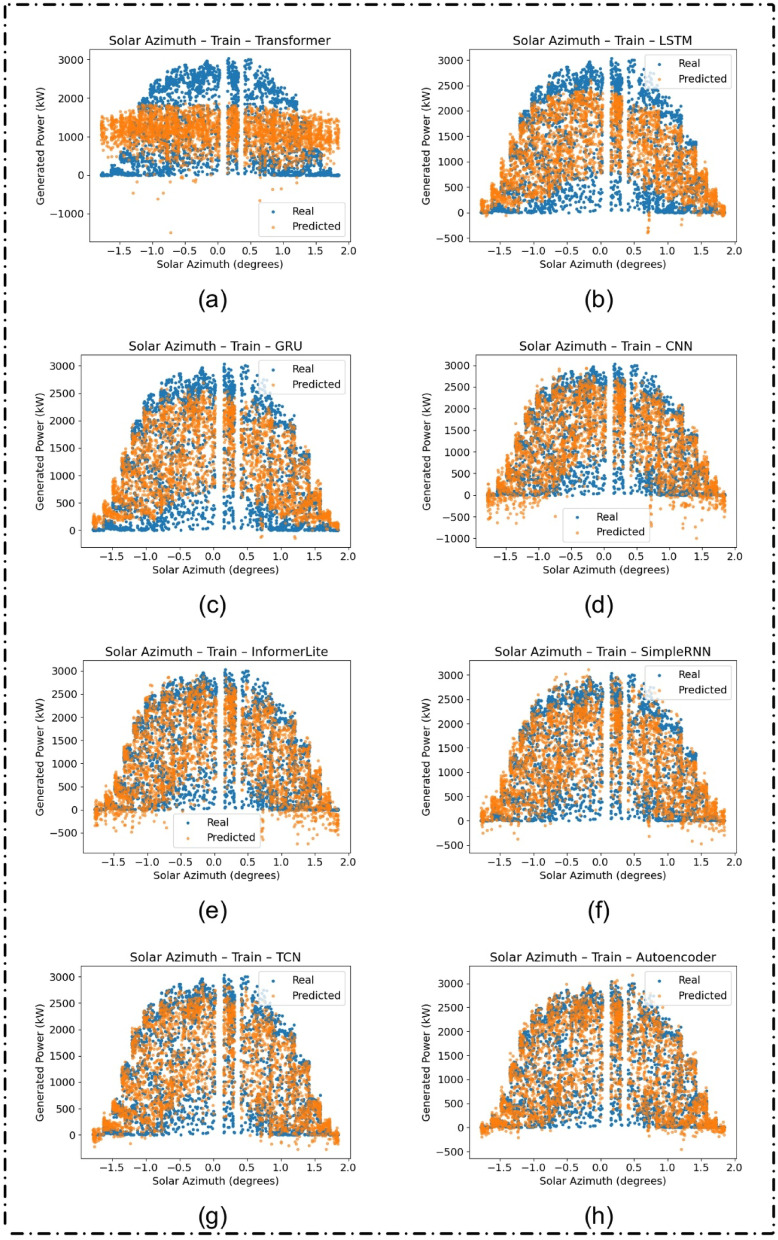
Generated versus predicted solar power (in kW) as a function of solar azimuth (in degrees) on the training dataset for different deep learning models: (**a**) Transformer, (**b**) LSTM, (**c**) GRU, (**d**) CNN, (**e**) InformerLite, (**f**) SimpleRNN, (**g**) Temporal Convolutional Network (TCN), and (**h**) Autoencoder.

**Fig. 9:**
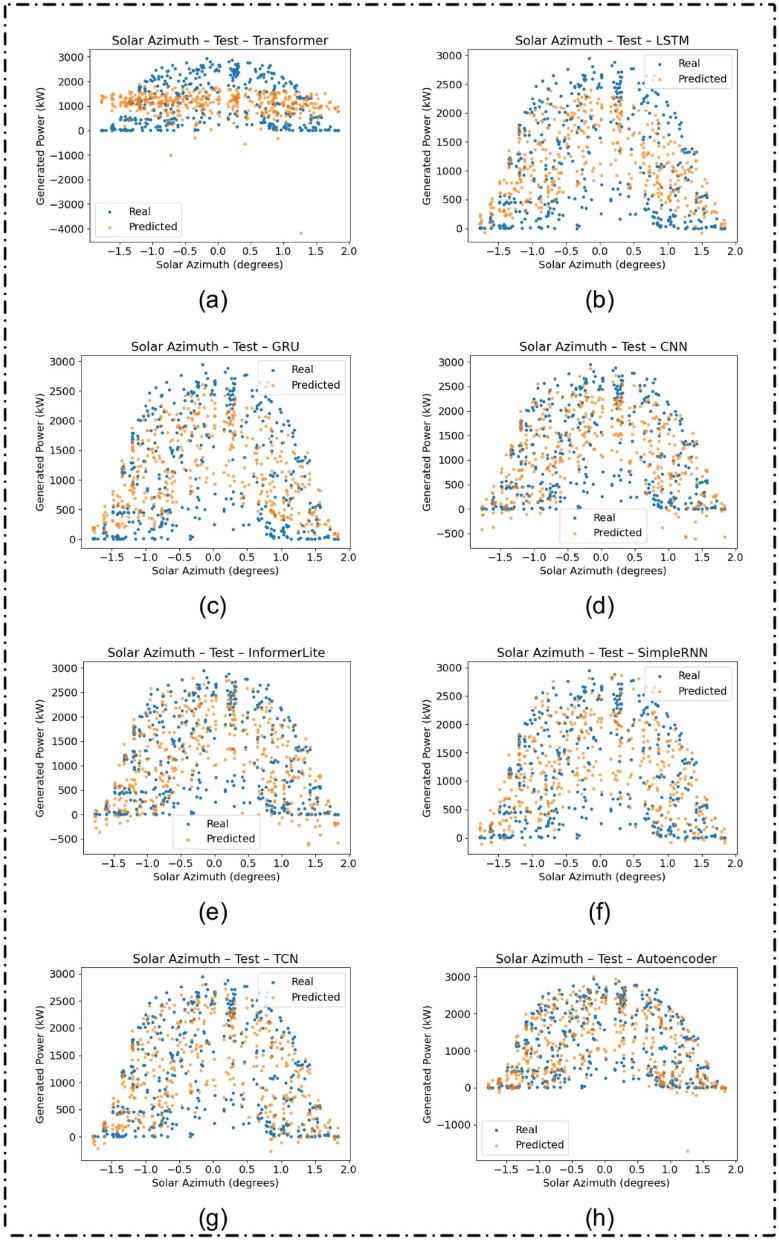
Generated versus predicted solar power (in kW) as a function of solar azimuth (in degrees) on the test dataset for different deep learning models: (**a**) Transformer, (**b**) LSTM, (**c**) GRU, (**d**) CNN, (**e**) InformerLite, (**f**) SimpleRNN, (**g**) Temporal Convolutional Network (TCN), and (**h**) Autoencoder.

Figures 8 and 9 analyze how model predictions vary with solar azimuth, a key feature in solar forecasting. Across both training and test sets, models like the Autoencoder and TCN consistently tracked the real values, reinforcing their robustness and capacity to incorporate temporal and directional features effectively.

### Residual error distribution

**Fig. 10:**
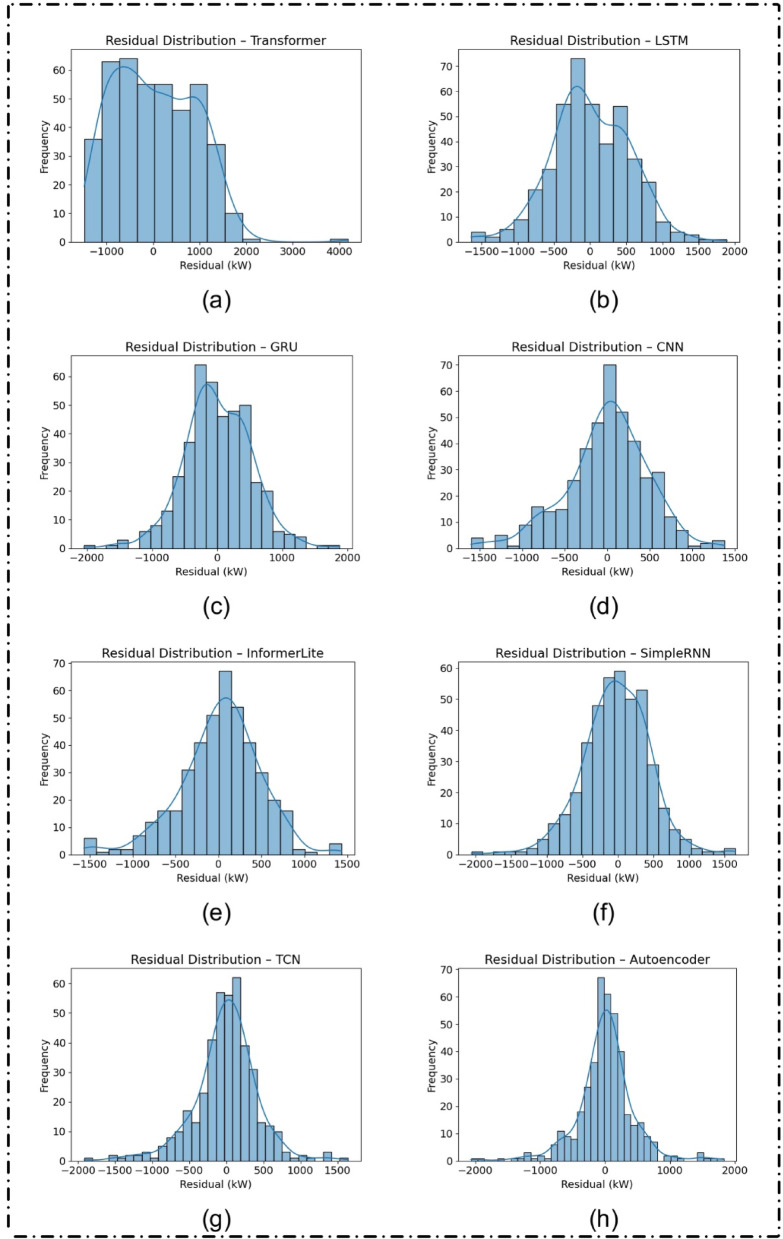
Residual error distributions (in kW) on the test dataset for different deep learning models, visualized with histograms and kernel density estimates: (**a**) Transformer, (**b**) LSTM, (**c**) GRU, (**d**) CNN, (**e**) InformerLite, (**f**) SimpleRNN, (**g**) Temporal Convolutional Network (TCN), and (**h**) Autoencoder.

The residual distributions shown in Fig. 10 provide further insights into model reliability. The Autoencoder and TCN had relatively symmetric and narrow error distributions, suggesting minimal bias and lower variance in predictions. The Transformer, on the other hand, exhibited a broader spread and signs of skewness, aligning with its poor test performance.

### Uncertainty Estimation with Monte Carlo dropout

**Fig. 11:**
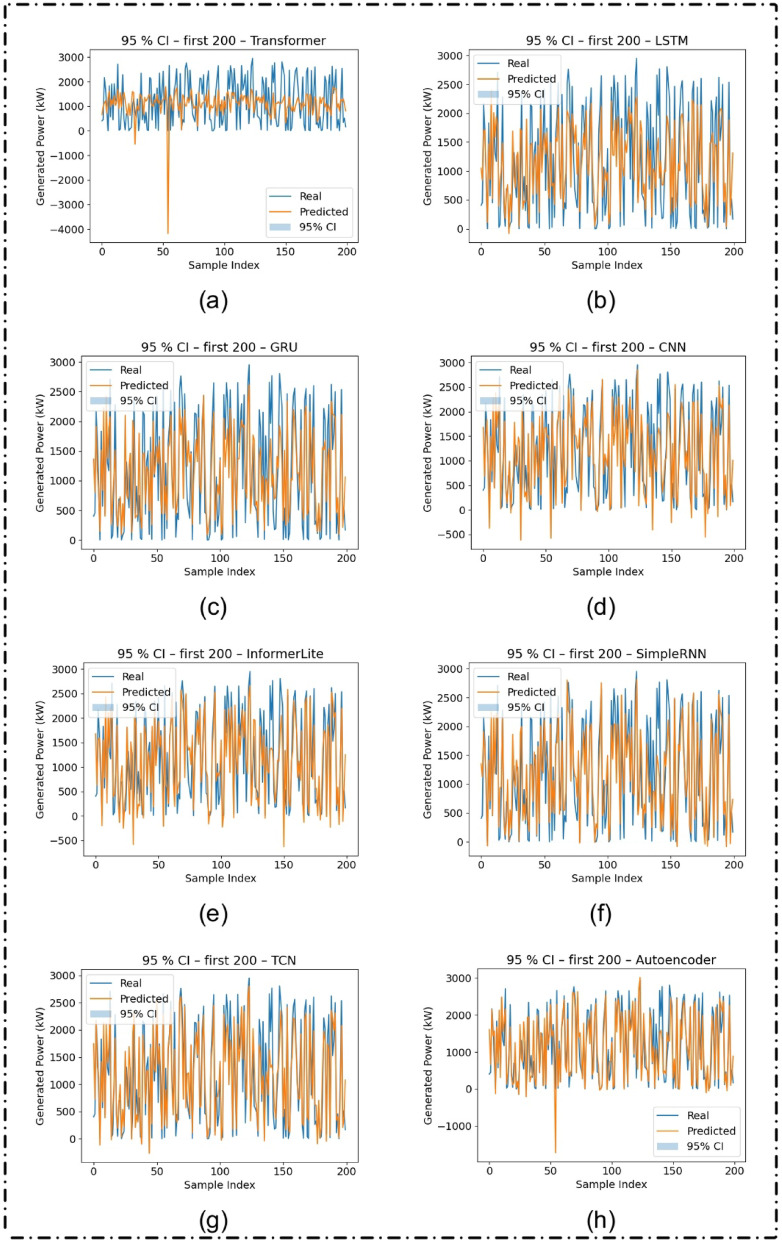
Predicted versus actual solar power generation (in kW) with 95% confidence intervals (CI) for the first 200 samples from the test dataset, using Monte Carlo dropout-based uncertainty estimation for different deep learning models: (**a**) Transformer, (**b**) LSTM, (**c**) GRU, (**d**) CNN, (**e**) InformerLite, (**f**) SimpleRNN, (**g**) Temporal Convolutional Network (TCN), and (**h**) Autoencoder.

Figure 11 shows that the 95% CIs produced with Monte Carlo dropout applied to the test predictions. The TCN and Autoencoder models come out on top in terms of providing accurate predictions and also relatively tight confidence bands, indicating high prediction confidence and robustness. The high degree of reliability is essential in real-world applications, for example in grid management of solar energy, where the uncertainty estimation has a significant impact for the operational decision process.

### Test set performance comparison

Table 5 presents test set performance, ordered by R². The TCN led with an R² of 0.7786, followed by the Autoencoder (0.7648) and SimpleRNN (0.7303). The Transformer again performed the worst (R² = 0.0714), affirming its unsuitability in this context without significant tuning or architectural adaptation.

**Table 5:** Test set performance of deep learning models ordered by increasing R² (Lower to higher Efficiency).

Model	RMSE	MAE	MAPE	*R*²
Transformer	879.6619	745.8583	35820.3200	0.0714
LSTM	543.2085	433.6328	23050.6910	0.6459
GRU	515.3331	403.0911	23191.7061	0.6813
CNN	482.4263	366.5691	28959.5578	0.7207
SimpleRNN	474.0869	362.3404	33187.8351	0.7303
InformerLite	475.2679	360.5724	20010.0381	0.7289
Autoencoder	442.6765	298.2939	18438.3318	0.7648
TCN	429.4863	306.5778	26614.8583	0.7786

### Extended model evaluation

Further insight into model behaviour is studied with other metrics, such as RSD and global R² over the entire dataset as shown in Table 6. The Autoencoder obtained the highest R² (Whole), overall (0.8437), indicating good performance over all partitions. In addition, the RSD of (RSD = 0.7499) was in reasonable limits between variability and accuracy.

**Table 6:** Extended performance metrics ordered by increasing R² on test set (R² - test).

Model	*R*² (Whole)	*R*² (Train)	*R*² (Test)	Mean (Test)	SD (Test)	RSD
Transformer	0.1506	0.1551	0.0714	1099.4890	455.0978	0.4139
LSTM	0.6800	0.6855	0.6459	1126.9376	617.3073	0.5478
GRU	0.7213	0.7307	0.6813	1130.9634	654.9133	0.5791
CNN	0.7474	0.7568	0.7207	1141.4810	766.4372	0.6714
InformerLite	0.7789	0.7958	0.7289	1116.9305	793.1338	0.7101
SimpleRNN	0.7894	0.8080	0.7303	1160.7808	722.1749	0.6221
Autoencoder	0.8437	0.8677	0.7648	1131.9559	848.8374	0.7499
TCN	0.8211	0.8374	0.7786	1147.5944	783.4791	0.6827

## Future work

Future studies should focus on the scalability, robustness, and adaptability of DL models for solar power prediction. One interesting bracket includes hybrid architectures that merge convolutional layers with temporal models such as LSTM or attention-based transformers. This kind of hibridization could allow for the simultaneous modeling of short-term characteristics and long-range dependence in solar power data. Similarly, ensemble methods such as model averaging or stacking, which could combine the power of different architectures and make more accurate and smoother predictions, are also worth investigating. Validation across geographic locations with varying climates would be needed to ensure the models generalize well across sites. This could be achieved with domain adaptation approaches or federated learning methodologies in order to diminish the necessity of remedial training on a per-site basis. Furthermore, include satellite-derived variables such as cloud drift, irradiance maps and atmospheric transparency from products like Geostationary Operational Environmental Satellite (GOES) or Himawari can be a great help for the model in order to address fast weather transitions. Future efforts will also explore extending the models to support multi-horizon forecasting, enabling predictions several hours ahead to better support grid operations and energy storage management. Lastly, optimizing the computational efficiency of models—especially those based on transformers—will be important for enabling real-time deployment in embedded systems or edge computing environments, where resource constraints are a key consideration.

## Conclusion

This study evaluated a set of advanced DL models—including RNN variants (SimpleRNN, GRU, LSTM), CNN, Transformer, Informer, Autoencoder, and TCN —for the task of solar power forecasting using a diverse range of meteorological and solar positional features. The TCN architecture achieved the best predictive performance according to performance metrics in general, particularly on the test data, which reflected its strong capacity for capturing the significant temporal patterns in an organized, robust and general manner. The Autoencoder model also exhibited good performance across the board, as marginally the best sequence-based model and in terms of clustering representing low-dimensional temporal features. On the other hand, the Transformer model did much worse than anticipated, by achieving the lowest R² on the test data, and the findings suggest it may not be suitable for this forecasting task given the input feature set and length of sequence, at least. GRU and LSTM based models had moderate success but appeared to underfit to longer dependencies without the presence of further attention mechanisms or architectural modifications. Reasonable level of the test performance on InformerLite and CNN’s better to capture short-term and mid-term of the temporal dependency. Nevertheless, this study is not without limitations. This dataset is geographically limited, and the generalization of these models in different climatic zones or terrains may be limited. The experiments only consider the next single time step prediction and do not encompass the multi-horizon complexity that is relevant to energy planning and control. Additionally, no satellite imagery or real-time sky state information are included in the model inputs, which could be useful to enhance forecast accuracy under overcast conditions. Overall, the models exhibit very strong performance in the present settings but more improvements and wider testing are necessary for deployment in practice.

## Data Availability

The datasets used and/or analysed during the current study available from the corresponding author on reasonable request.

## Abbreviations

Abbreviation: Full Form
AI: Artificial Intelligence
ANN: Artificial Neural Network
API: Application Programming Interface
ARIMA: AutoRegressive Integrated Moving Average
CI: Confidence Interval
CNN: Convolutional Neural Network
DL: Deep Learning
DNN: Deep Neural Network
EDA: Exploratory Data Analysis
EEMD: Ensemble Empirical Mode Decomposition
EV: Electric Vehicle
GBM: Gradient Boosting Machine
GOES: Geostationary Operational Environmental Satellite
GRU: Gated Recurrent Unit
InformerLite: Lightweight Informer for Long Sequence Time-Series Forecasting
LightGBM: Light Gradient Boosting Machine
LSTM: Long Short-Term Memory
MAE: Mean Absolute Error
MAPE: Mean Absolute Percentage Error
ML: Machine Learning
MSE: Mean Squared Error
PV: Photovoltaic
RELAD-ANN: Regularized Lightweight Artificial Neural Network
RF: Random Forest
RMSE: Root Mean Squared Error
RNN: Recurrent Neural Network
RSD: Relative Standard Deviation
R²: Coefficient of Determination
SD: Standard Deviation
SEFMNN: Stack-based Ensemble Fusion with Meta-Neural Network
SimpleRNN: Simple Recurrent Neural Network
TCN: Temporal Convolutional Network
TIR: Transformer-Infused Recurrent Neural Network
XGB-SE: Extreme Gradient Boosting - Stacked Ensemble
